# Mirror neuron activity depending on the content and stage of the observed action: a TMS study

**DOI:** 10.55730/1300-0144.5710

**Published:** 2023-08-11

**Authors:** H. Evren BORAN, Hasan KILINÇ, Bülent CENGİZ

**Affiliations:** 1 Department of Neurology and Division of Clinical Neurophysiology, Faculty of Medicine, Gazi University, Ankara, Turkiye; 2 Neuropsychiatry Center, Gazi University, Ankara, Turkiye; 3 Neuroscience and Neurotechnology Center of Excellence, Ankara, Turkiye

**Keywords:** Mirror neuron system, transcranial magnetic stimulation, motor evoked potential, repeated action observation

## Abstract

**Background/aim:**

The firing rate of the mirror neuron system in monkeys decreases systematically with more repetitions. The aim of this study is to investigate whether the activity of the mirror neuron system varies based on the observed movement and the contents of the action, as well as whether there is inhibition in the mirror neuron system when humans observe repeated actions. If inhibition is present, the second question of the study is whether it is related to the organization of the observed action.

**Materials and methods:**

Fourteen healthy volunteers participated in the study. Transcranial magnetic stimulation was applied to the left primary motor cortex and motor evoked potentials (MEPs) were recorded from the right first dorsal interosseous and abductor pollicis brevis muscles while the participants were watching videos specially prepared for the study.

**Results:**

There were no significant changes in MEP amplitudes compared to baseline MEPs while observing aimless action. However, while participants watched the repeated action video, the mean MEP amplitude increased at the beginning of the movement, but neither facilitation nor inhibition was detected when the participants watched the phase of grasping the object of the action compared to the baseline MEP amplitude. On the other hand, while participants were watching different activities, an increased MEP amplitude was observed at the beginning of the movement and in the grasping of the object of the action. Additionally, there was no significant reduction in MEP amplitude during any movement stages while observing the repeated action video.

**Conclusion:**

The findings of this study suggest that the activation of the mirror neuron system in humans depends on the content and stages of the observed movement. Additionally, there was no inhibition or systematic reduction in MEP amplitudes while watching a repeated action.

## 1. Introduction

The mirror neuron system (MNS) entails the activity of the neuron population that is activated both when an individual performs a specific action and when individuals observe a similar action performed by another individual. Mirror neurons were first discovered in ventral premotor area F5 of the macaque monkey [1]. They were also shown to exist in a region of the inferior parietal lobule [2]. Human neuroimaging studies showed activation in homologous cortical areas when individuals observed and performed the same actions [3,4]. Furthermore, a cortical recording study of macaque monkeys found that in addition to area F5, the hand area of the motor cortex exhibited mirror-like properties [5].

A study demonstrated that neurons in the F5 area of the macaque monkey predominantly discharged during the observation of purposeful actions but did not fire while the monkey was watching mimicked or pantomimed actions [6]. The same study showed that a subset of F5 neurons discharged while monkeys observed the final stages of movement directed toward an object but not when the same motion was performed without an object [6]. Kohler et al. found that a part of the mirror neurons became active while observing action accompanied by sounds and was also activated by sound alone in monkeys [7]. Mirror neuron activity plays a role in comprehending the goal of motor actions being done by a person at that moment [8].

In humans, a study showed that observing hand movements with an object activated the ventral premotor cortex and posterior parietal lobe areas, while the imitation of the same actions in the absence of objects only activated the premotor area [9]. Both action observation and imitation activated the inferior frontal gyrus [10]. Additionally, action planning, execution, and observation tasks activated the intraparietal sulcus [11,12]. The inferior frontal gyrus and intraparietal sulcus comprise the human MNS for action representation [13].

In the monkey brain, repeating the same stimulus causes a decrease in neuronal firing [14]. In adult humans, the repetition of a stimulus usually results in a reduction of the blood oxygen level signal in the relevant brain regions. This phenomenon is known as repetition suppression and has been observed across various domains, including numbers [15], objects [16], and semantics [17].

Caggiano et al. demonstrated that F5 neurons in monkeys did not adapt to observations of repeated actions. While they detected a decrease in the response of a small number of F5 neurons and an increase in a few others, overall the neurons did not change their firing in response to the observation of repeated actions [18]. Kilner et al. showed no significant difference in mirror neuron firing rates from the first to the second presentation. However, they showed that the firing rate systematically decreased with more repetitions of the same action [19].

In humans, Hamilton et al. showed that repeated observation of the same goal action led to a systematic reduction of activation in the left intraparietal sulcus. However, they found that repeated observation of the same hand trajectory did not cause suppression [20].

This study explores whether MNS activity changes according to the observed movement and the movement’s contents. In addition, this study aims to test the hypothesis that repeated observation will suppress MNS activity in humans. To achieve these goals, we prepared different videos representing various characteristics of movement in humans.

## 2. Materials and methods

### 2.1. Study population

Fourteen healthy right-handed individuals (4 women, 10 men) aged 24–48 years (mean age: 32 years) without systemic or neurological diseases were enrolled in this study. All participants provided written informed consent, and the Ankara Numune Training and Research Hospital Clinical Research Ethics Committee (E-Kurul-E-14-289) approved the study.

### 2.2. Videos

**Figure 1:**
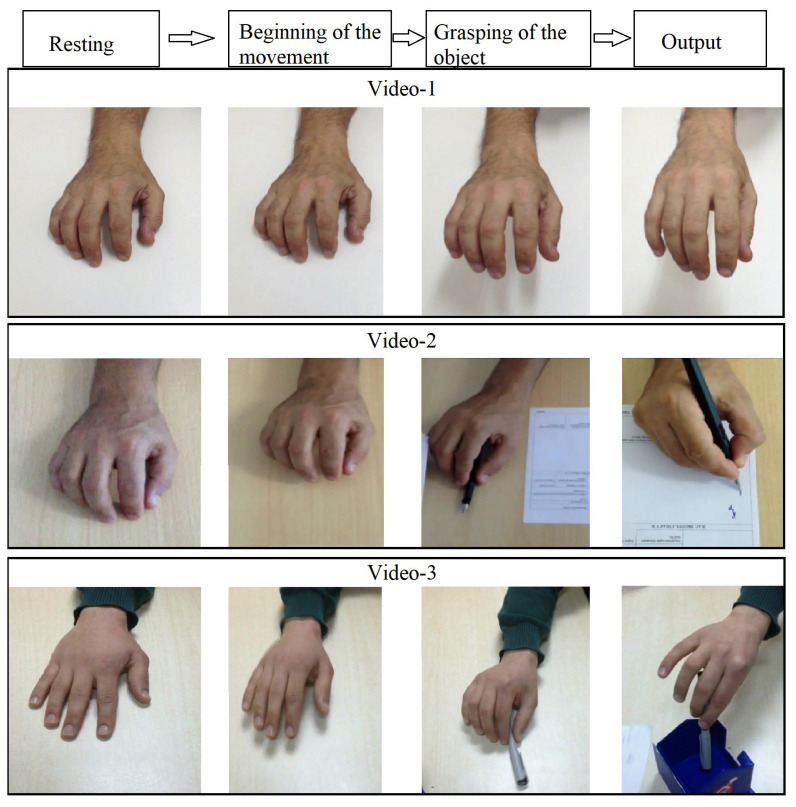
Sequences of each video’s contents. Video-1 consisted of 15 stereotypical movements in which a right hand was resting on a table and then lifted aimlessly from the table. Video-2 consisted of 15 repetitive movements involving reaching out to a pen, grasping the pen, and writing on paper. Video-3 consisted of 15 various activities: reaching out, gripping the pen, and different motor outputs, such as putting the pen in a pen holder, closing the cap, etc. Transcranial magnetic stimulations were applied at the beginning of the movement and the grasping of the object.

The study involved having the volunteers watch three different videos with intervals of at least 1 week between each viewing. Video-1 consisted of 15 stereotypical movements of a right hand resting on a table and lifted aimlessly from the table. Video-2 featured a pen as an object, and the aim was to reach the pen, grasp it, and write on paper. This video consisted of 15 repetitive movements. Video-3 involved different actions and movements with the pen as an object, featuring 15 different activities, such as putting the pen in a pen holder, closing the cap, etc., after the hand was resting on the table, grasping the pen. After adjusting the video sequences, the beginning of the movement (BM) and the grasping of the object (GO) were fixed. There were 15 sequences in each video, and each sequence was about 13.6 s. Each video length was about 4 min. Figure 1 shows the contents of the videos.

The volunteers were asked to watch the video on a screen 100 cm away while sitting in a comfortable chair. Participants were asked to watch the videos carefully and avoid imagining the observed actions.

### 2.3. Transcranial magnetic stimulation (TMS) procedure

Single-pulse stimulations were applied over the hand area of the left motor cortex using a figure-8-shaped coil with a diameter of 7 cm, connected to a Magstim 200 single-pulse stimulator (Magstim Co. Ltd., Whitland, Wales). The coil was oriented to induce current flow in a posterior-anterior direction. Electromyography (EMG) recordings were obtained from the right first dorsal interosseous (FDI) muscle and the right abductor pollicis brevis (APB) muscle using 10-mm Ag-AgCl surface recording electrodes. The coil was adjusted to an optimal position for obtaining a motor evoked potential (MEP) from the FDI muscle. A cap was placed on the scalp and the optimal position of the coil was marked, and the position of the coil was continuously checked during the experiment. EMG signals were filtered (30–3000 Hz) and digitized at a sample rate of 10,000 Hz using a Digitimer D440 4-channel isolated amplifier (Digitimer, Hertfordshire, UK) and recorded with a CED-1401 analog-to-digital converter (Cambridge Electronic Design, Cambridge, UK). KMPlayer software was used to play the video, and synchronization between KMPlayer and Signal 4.0 was achieved with Auto Mouse Clicker software [21]. Offline analysis of the signals was performed using Signal Software (Cambridge Electronic Design, UK).

Each volunteer was required to participate in the study three times with intervals of at least 1 week between sessions. In each session, the magnetic stimulation intensity was adjusted to elicit MEPs with an amplitude of approximately 1 mV in the FDI muscle. After determining the threshold of the 1-mV MEP, baseline MEP amplitudes were obtained by averaging the values of 15 MEPs that resulted from stimulation of the contralateral motor cortex at the same stimulation intensity with a fixed rate of 0.2 Hz during the resting state of the muscles. Following the baseline MEP measurements, volunteers were instructed to watch the video in a relaxed manner, while MEPs were recorded during the watching of movements at the BM and GO stages using the same stimulation intensity. MEP amplitudes were calculated individually as the average value of 15 MEPs at each stage and measured as the peak-to-peak amplitude. Videos were randomly assigned to participants in each session.

### 2.4. Statistical analysis

All data analyses were conducted using IBM SPSS Statistics 22.0 for Windows (IBM Corp., Armonk, NY, USA). Baseline MEP amplitudes were compared with the MEP amplitudes obtained while the participants watched the BM and GO stages on the screen. Repeated ANOVA with the factors of “TMS timing” in terms of the timeline of the movement on the screen (baseline × BM × GO) and “recording muscle” (FDI × APB) was performed for each video result separately. The distribution of the data was checked with the Shapiro–Wilk test. Values of P < 0.05 were considered statistically significant. In order to detect Cohen’s medium effect size of f = 0.025 (1) with 80% power in repeated measures, within-factors ANOVA (three groups, alpha = 0.05) performed with G*Power 2 suggested that we would need 12 participants (N = 12) [22].

## 3. Results

Eight out of the 14 volunteers participating in the study watched all three videos. Ten volunteers watched the first video, 11 volunteers watched the second video, and 11 volunteers watched the third video.

### 3.1. Effect of watching videos on MEP amplitude

**Figure 2:**
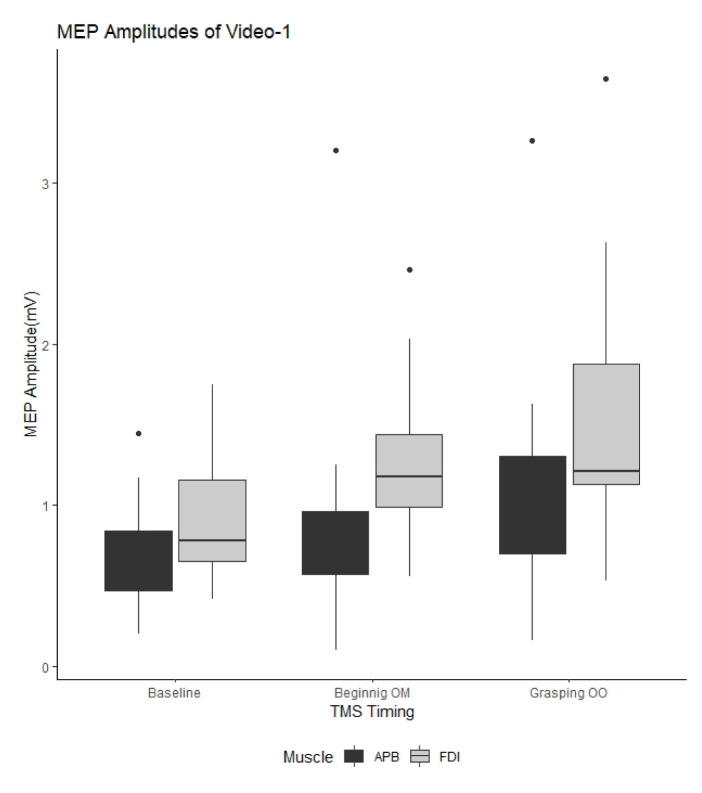
Comparison of motor evoked potential amplitudes in Video-1. MEP: Motor evoked potential; Beginning OM: beginning of the movement; Grasping OO: grasping of the object; TMS: transcranial magnetic stimulation; APB: abductor pollicis brevis; FDI: first dorsal interosseous.

**Figure 3:**
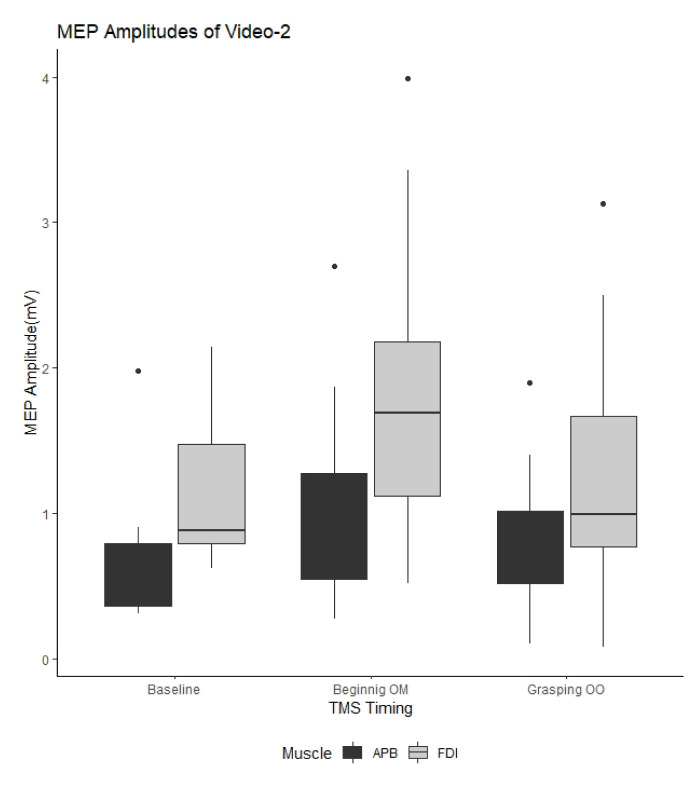
Comparison of motor evoked potential amplitudes in Video-2. MEP: Motor evoked potential; Beginning OM: beginning of the movement; Grasping OO: grasping of the object; TMS: transcranial magnetic stimulation; APB: abductor pollicis brevis; FDI: first dorsal interosseous.

**Figure 4:**
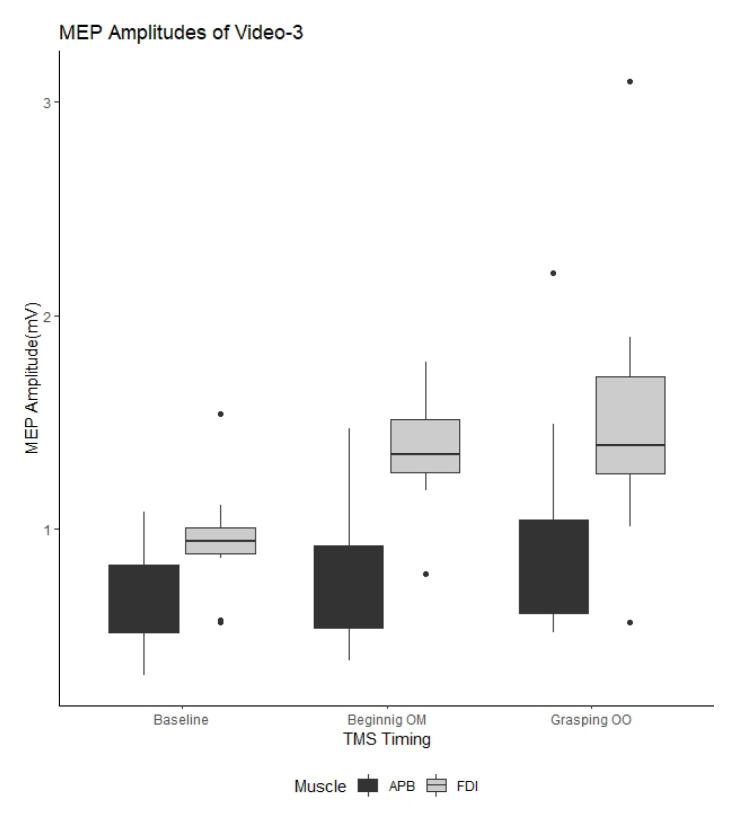
Comparison of motor evoked potential amplitudes in Video-3. MEP: Motor evoked potential; Beginning OM: beginning of the movement; Grasping OO: grasping of the object; TMS: transcranial magnetic stimulation; APB: abductor pollicis brevis; FDI: first dorsal interosseous.

Figures 2–4 show the mean MEP amplitudes obtained for each video. In addition, the ANOVA results regarding the watched videos are presented in the Table. The ANOVA results showed that the factor of “recording muscle” had the main effect on MEP amplitude for all videos, while the effect of “TMS timing” on MEP amplitude was detected for Video-2 and Video-3. Moreover, significant “TMS timing” × “recording muscle” interaction was detected from the data obtained for Video-2 and Video-3. Therefore, we conducted separate analysis of the effect of “TMS timing” based on the recording muscle.

For Video-1, “TMS timing” had no effect on MEP amplitude [F(2, 20) = 2.1, P = 0.15] (Figure 2).

For Video-2, statistical analysis results for the FDI muscle revealed that the MEPs obtained when the participants observed the BM stage were significantly higher than the baseline MEP amplitudes (P = 0.001). There was no significant difference between the MEPs obtained during the GO stage and baseline MEP amplitudes (P > 0.05). The mean MEP amplitude obtained from the participants while they watched the BM part of the video’s movement was significantly higher than the mean MEP amplitude obtained while watching the GO for the FDI muscle (P = 0.003) (Figure 3). In addition, a significantly increased mean MEP amplitude for the BM of TMS timing was detected for the APB muscle compared to the baseline mean MEP amplitude (P = 0.02) (Figure 3).

For Video-3, the mean MEP amplitudes obtained for the BM and GO stages of TMS timing were significantly increased compared to the baseline MEP amplitudes for the FDI (P = 0.003 and P = 0.007, respectively) and APB muscles (P = 0.005 and P = 0.008, respectively) (Figure 4).

### 3.2. Comparison of observed action contents of the videos

To compare the effects of the video contents on MEP amplitude, we first calculated the MEP amplitude increase ratio as MEP amplitude obtained with BM TMS timing/baseline MEP amplitude (MEPBM/MEPbaseline) and MEP amplitude obtained with GO TMS timing/baseline MEP amplitude (MEPGO/MEPbaseline). ANOVA tests were designed to investigate the effects of “video” (“Video-1” × “Video-2” × “Video-3”) and “TMS timing” (“BM” × “GO”) on MEP amplitude for the FDI and APB muscles. The results showed no main effects of “video” or “TMS timing” on MEP amplitude for the FDI (F[0.7, 4.2] = 0.98, P = 0.4 and F[0.01, 0.5] = 0.72, P = 0.7, respectively) and APB (F[2.5, 15.1] = 0.98, P = 0.3 and F[0.2, 0.3] = 0.15, P = 0.1, respectively) muscles. However, there was a significant interaction effect between “TMS timing” and “video” for the FDI muscle (F[0.8, 1.3] = 4.1, P = 0.02). This finding indicated that “TMS timing” had different effects on mean MEP amplitudes depending on the observed video type for the FDI muscle. The Bonferroni corrected t-test showed that watching the GO stage resulted in a significantly greater MEP amplitude increase in Video-2 than in Video-1 (P = 0.002), while there was no difference in BM-related MEP facilitation regarding watching the videos (P > 0.05).

### 3.3. MEP distribution for the same object and same aim

Watching Video-2 resulted in an increased MEP amplitude during the BM stage compared to the baseline, while neither facilitation nor inhibition was detected during GO compared to the baseline MEP amplitude. We explored the time course of MEP amplitude changes during both the BM and GO stages of the observed movement to investigate this discrepancy in Video-2. For this, 15 MEP values obtained from healthy subjects were divided into three parts: the first five (1–5) MEPs (MEP_1-5_), middle five (6–10) MEPs (MEP_6-10_), and last five (11–15) MEPs (MEP_11-15_). Mean values for MEP_1-5_, MEP_6-10_, and MEP_11-15_ were calculated. Repeated ANOVA testing indicated that there was no significant difference between the mean values of MEP_1-5_, MEP_6-10_, or MEP_11-15_ in the BM or GO stages obtained from the FDI and APB muscles (P > 0.05).

## 4. Discussion

The main results of this study were as follows: First, no significant MEP amplitude changes were detected while the participants were watching the objectless and aimless video; however, MEP amplitudes increased while they watched a video that contained an object and goal-directed movements. Second, while the participants were watching the beginning of the movement action, the mean MEP amplitude significantly increased in all videos that contained object and goal-directed movements. Third, watching the video that contained different goals and actions of grasping the object significantly increased the MEP amplitudes. Fourth, while watching a repeated action, observing the beginning of the movement action significantly increased the MEP amplitude. Finally, neither inhibition nor systematic reduction was observed while participants watched a repeated action.

Our first finding of MEP amplitude increasing while participants watched another person’s action including an object and goal is consistent with previous TMS studies [23–25]. One study showed that corticospinal excitability increased in recordings from the tibialis anterior and soleus muscles during gait observation [26]. Another study found MEP facilitation during observations of direct movement [27]. These studies revealed that MEP amplitudes increased when participants observed movements. Rens et al. detected that observation of the lifting of an object increased corticospinal excitability regarding the object’s weight [28].

Most evidence suggests that during action observation, the primary motor cortex (M1) is the source of modulation in corticospinal excitability. It has been concluded that the mechanism that causes changes in MEP amplitude during action observation is the activation of the premotor and parietal areas, followed by the generation of representation of the observed movement in M1 through cortico-cortical connections [29–31]. The increase of MEP amplitudes during action observation appears to be phase-dependent. The most significant increase in MEP amplitude occurs when specific muscles contract during actual task performance [25]. However, Syrov et al. found no increase in MEP amplitudes in natural and unnatural action observations. They suggested that their TMS timing may not have led to an increase in MEP amplitudes due to the phase dependence of activation [32]. In our study, we found increased MEP amplitudes while the participants observed the beginning of purposeful movement action in the presence of an object and while observing the grasping of the object for different aims. The most significant increase in MEP amplitude was observed while participants watched the video that contained the gripping of the object for distinct goals. This finding may indicate that the key factor is the goal’s presence while watching other people’s actions regarding MNS activation. It was shown that adults encode actions in terms of their outcomes [33]. Humans interpret basic movements in terms of the actors’ aims and intentions while observing others’ actions. In particular, aims are the main factor for action planning and our interpretations of other people’s actions. Fogassi et al. showed that single cells in the inferior parietal lobe responded selectively to both the execution and observation of an action within a sequence leading to a specific goal in monkeys. However, they did not respond to the same action when it was part of a sequence reaching a different purpose [34].

No significant changes were observed while watching the video containing neither object nor purpose. The relationship between the video contents and the recording muscles may explain this finding. This video showed a right hand being lifted from a table without a grasping action and the hand muscles were inactive. The recordings were made for the APB and FDI muscles. The MNS becomes active during the observation of actions of relevant body parts. Cavallo et al. conducted a reverse pliers experiment in humans using TMS. MEPs were recorded from the first dorsal interosseous and the opponens pollicis muscles while observing and executing grip movements with classical and reverse pliers. Their study results indicated that motor cortex excitability reflects the configurative relationship between body parts in observed hand movements. They suggested the importance of encoding the configural body part relationships involved in the action rather than the aim of the action when individuals are observing another person performing a task with pliers [23]. Thenar muscles (e.g., the abductor pollicis brevis muscle) are used for writing in humans [35]. In our study, we found increased MEP amplitudes in the APB muscle. The object was a pen in both the video with the same object and the same goal and the video with the same object and a different goal. Most people think of writing as the first action to come to mind when a pen is seen on the screen, which may lead to an increase in MEP amplitude for the APB muscle.

In this study, participants were not informed about the videos they would watch before the experiment started, nor were they aware of whether they would watch repeated actions. Participants may have expected the movement to be different each time they watched the repeated action video, and they may have also anticipated that the same action would not occur at the beginning of the movement. This may have resulted in an increase in MEP amplitudes at the beginning of the movement but not during the grasping of the object. Kilner et al. did not find a significant change in the firing rate of mirror neurons from the first to the second presentation in observations of a repeated action. Their study showed that the firing rate of F5 neurons in monkeys decreased systematically with more repetitions [19]. In contrast, Caggiano et al. demonstrated that F5 neurons were not suppressed while monkeys observed repeated actions. The activity of a small number of F5 neurons decreased while the firing rate of a few F5 neurons increased, and most of the recorded single neurons did not show a significant reduction in firing rate [18]. Kuravi et al. found that macaque superior temporal sulcus neurons, which are indirectly connected to F5, showed repetition suppression in the action phase in the study of single and multiunit recordings [36]. Watching the grasping of an object by hand is a complex stimulus that involves the simultaneous representation of two different stimuli, namely the hand and the object. The form of the hand varies while at rest, at the beginning of the movement, and during the grasping action. Repetition of the action phase (i.e., the approaching and reaching stage) can be interrupted by another action phase [36]. This could explain why we did not observe any inhibition in our participants while they watched repeated action. On the contrary, MEP amplitudes increased while they watched the beginning of the movement compared to the baseline MEP amplitude. Furthermore, we did not detect any systematic inhibition in our study. Hamilton et al. showed a systematic reduction of activation in the left intraparietal sulcus while participants watched repeated actions directed at the same aim. However, no suppression was detected upon repeated observations of the same trajectory in this region [20]. In our study, the participants watched 15 identical sequences in a repeated action video. Hence, the trajectory and movements in the repeated action video were always the same. Kilner et al. showed that inhibition in the firing rate with repetition occurred for both facilitation and inhibition mirror neurons [19]. They detected that facilitation mirror neurons were less active and the suppression mirror neurons were more inhibited, leading to no significant changes at the population level in monkeys [19]. Our study with TMS did not examine the subgroups of the MNS separately but rather revealed the overall output. MEP amplitude was neither inhibited nor facilitated while participants watched the sequence of the grasping of an object in the repeated action video. This finding can be considered consistent with the results of other studies [19,20].

From a clinical perspective, many studies have reported successful results using mirror therapy in the treatment of some neurological diseases such as stroke and phantom pain [37–43]. Our study results suggest that it may be more beneficial to perform different movements rather than repeating the same movements during mirror therapy sessions used in the rehabilitation process of some neurological diseases when combined.

This study has some limitations. First, only 8 out of 14 healthy volunteers watched all videos in this study. Second, the participants were encouraged to watch carefully without thinking or imagining the action, but the participants’ attention was not measured while they watched the videos. Finally, recordings were made from the APB and FDI muscles. The aimless and objectless video contained a sequence of a hand rising from a table without hand muscle activation, and no activation of the FDI and APB muscles might be expected in that movement. Therefore, activation may not have been detected in these muscles while watching this video. At the same time, the study has some strengths, as evaluations were performed while participants observed the same or different aims in different stages. Studies that vary the objects presented could provide more information on this topic.

In conclusion, the MNS was evaluated while participants watched actions with or without an object and aim. Furthermore, it was assessed with both repeated and different movements in this study. This study has demonstrated that a significant increase in MEP amplitude occurs while watching a video containing an object and aim. Watching the beginning of the movement action significantly increased corticomotor excitability while participants were viewing repeated action. This may have arisen from the expectations of the participants. Inhibition or systematic reduction in MEP amplitudes was not observed while they watched repeated action.

## Figures and Tables

**Table:** Results of ANOVA regarding the watched videos.

		F	P
Video 1	TMS timing	(2, 20) = 2.1	0.15
	Muscle	(1, 10) = 37.16	0.000
	TMS timing × Muscle	(2, 20) = 0.91	0.42
Video 2	TMS timing	(2, 22) = 10.9	0.001
	Muscle	(1, 11) = 8.56	0.014
	TMS timing × Muscle	(2, 22) = 4.24	0.047
Video 3	TMS timing	(1, 10) = 25.01	0.001
	Muscle	(1, 9) = 14.89	0.004
	TMS timing × Muscle	(2, 20) = 6.89	0.005

TMS: Transcranial magnetic stimulation.

## References

[1] Di Pellegrino G, Fadiga L, Fogassi L, Gallese V, Rizzolatti G (1992). Understanding motor events: a neurophysiological study. Experimental Brain Research.

[2] Fogassi L, Gallese V, Fadiga L, Rizzolatti G Neurons responding to the sight of goal directed hand/arm actions in the parietal area PF (7b) of the macaque monkey.

[3] Rizzolatti G, Fadiga L, Matelli M, Bettinardi V, Paulesu E (1996). Localization of grasp representations in humans by PET: 1. Observation versus execution. Experimental Brain Research.

[4] Decety J, Grezes J, Costes N, Perani D, Jeannerod M (1997). Brain activity during observation of actions. Influence of action content and subject’s strategy. Brain.

[5] Kraskov A, Philipp R, Waldert S, Vigneswaran G, Quallo MM (2014). Corticospinal mirror neurons. Philosophical Transactions of the Royal Society of London Series B, Biological Sciences.

[6] Umilta MA, Kohler E, Gallese V, Fogassi L, Fadiga L (2001). I know what you are doing. A neurophysiological study. Neuron.

[7] Kohler E, Keysers C, Umiltà MA, Fogassi L, Gallese V (2002). Hearing sounds, understanding actions: action representation in mirror neurons. Science.

[8] Rizzolatti G, Fogassi L, Gallese V (2001). Neurophysiological mechanisms underlying the understanding and imitation of action. Nature Reviews Neuroscience.

[9] Buccino G, Binkofski F, Fink G, Fadiga L, Fogassi L (2001). Action observation activates premotor and parietal areas in a somatotopic manner: an fMRI study. European Journal of Neuroscience.

[10] Iacoboni M, Woods RP, Brass M, Bekkering H, Mazziotta JC (1999). Cortical mechanisms of human imitation. Science.

[11] Frey SH, Vinton D, Norlund R, Grafton ST (2005). Cortical topography of human anterior intraparietal cortex active during visually guided grasping. Cognitive Brain Research.

[12] Grezes J, Decety J (2001). Functional anatomy of execution, mental simulation, observation, and verb generation of actions: a meta-analysis. Human Brain Mapping.

[13] Rizzolatti G, Craighero L (2004). The mirror-neuron system. Annual Review of Neuroscience.

[14] Miller EK, Gochin P, Gross CG (1991). Habituation-like decrease in the responses of neurons in inferior temporal cortex of the macaque. Visual Neuroscience.

[15] Naccache L, Dehaene S (2001). The priming method: imaging unconscious repetition priming reveals an abstract representation of number in the parietal lobes. Cerebral Cortex.

[16] Grill-Spector K, Malach R (2001). fMR-adaptation: a tool for studying the functional properties of human cortical neurons. Acta Psychologica.

[17] Thompson-Schill SL, D’Esposito M, Kan IP (1999). Effects of repetition and competition on activity in left prefrontal cortex during word generation. Neuron.

[18] Caggiano V, Pomper JK, Fleischer F, Fogassi L, Giese M (2013). Mirror neurons in monkey area F5 do not adapt to the observation of repeated actions. Nature Communications.

[19] Kilner JM, Kraskov A, Lemon RN (2014). Do monkey F5 mirror neurons show changes in firing rate during repeated observation of natural actions?. Journal of Neurophysiology.

[20] Hamilton AF, Grafton ST (2006). Goal representation in human anterior intraparietal sulcus. Journal of Neuroscience.

[21] Cengiz B, Vurallı D, Zinnuroğlu M, Bayer G, Golmohammadzadeh H (2018). Analysis of mirror neuron system activation during action observation alone and action observation with motor imagery tasks. Experimental Brain Research.

[22] Faul F, Erdfelder E, Lang AG, Axel B (2007). G*Power 3: A flexible statistical power analysis program for the social, behavioral, and biomedical sciences. Behavior Research Methods.

[23] Cavallo A, Becchio C, Sartori L, Bucchioni G, Castiello U (2012). Grasping with tools: corticospinal excitability reflects observed hand movements. Cerebral Cortex.

[24] Fadiga L, Fogassi L, Pavesi G, Rizzolatti G (1995). Motor facilitation during action observation: a magnetic stimulation study. Journal of Neurophysiology.

[25] Gangitano M, Mottaghy F, Pascual-Leone A (2001). Phase-specific modulation of cortical motor output during movement observation. NeuroReport.

[26] Ito T, Tsubahara A, Shiraga Y, Yosuke Y, Daisuke K (2020). Motor activation is modulated by visual experience during cyclic gait observation: a transcranial magnetic stimulation study. PLoS One.

[27] Prinsen J, Alaerts K (2020). Enhanced mirroring upon mutual gaze: multimodal evidence from TMS-assessed corticospinal excitability and the EEG mu rhythm. Scientific Reports.

[28] Rens G, van Polanen V, Botta A, Gann MA, Orban de Xivry JJ (2020). Sensorimotor expectations bias motor resonance during observation of object lifting: the causal role of pSTS. Journal of Neuroscience.

[29] Baldissera F, Cavallari P, Craighero L, Fadiga L (2001). Modulation of spinal excitability during observation of hand actions in humans. European Journal of Neuroscience.

[30] Hari R, Forss N, Avikainen S, Veskari E, Salenius S (1998). Activation of human primary motor cortex during action observation: a neuromagnetic study. Proceedings of the National Academy of Sciences of the United States of America.

[31] Strafella AP, Paus T (2000). Modulation of cortical excitability during action observation: a transcranial magnetic stimulation study. NeuroReport.

[32] Syrov N, Bredikhin D, Yakovlev L, Miroshnikov A, Kaplan A (2022). Mu-desynchronization, N400 and corticospinal excitability during observation of natural and anatomically unnatural finger movements. Frontiers in Human Neuroscience.

[33] Hommel B, Musseler J, Aschersleben G, Prinz W (2001). The theory of event coding (TEC): a framework for perception and action planning. Behavioral and Brain Sciences.

[34] Fogassi L, Ferrari PF, Gesierich B, Rozzi S, Chersi F (2005). Parietal lobe: from action organization to intention understanding. Science.

[35] Naider-Steinhart S, Katz-Leurer M (2007). Analysis of proximal and distal muscle activity during handwriting tasks. American Journal of Occupational Therapy.

[36] Kuravi P, Caggiano V, Giese M, Vogels R (2016). Repetition suppression for visual actions in the macaque superior temporal sulcus. Journal of Neurophysiology.

[37] Won SH, Kim JC, Oh DW (2015). Effects of a novel walking training program with postural correction and visual feed-back on walking function in patients with post-stroke hemiparesis. Journal of Physical Therapy Science.

[38] Ezendam D, Bongers R, Jannink MJA (2009). Systematic review of the effectiveness of mirror therapy in upper extremity function. Disability and Rehabilitation.

[39] Arya KN, Pandian S, Kumar D, Puri V (2015). Task-based mirror therapy augmenting motor recovery in poststroke hemiparesis: a randomized controlled trial. Journal of Stroke and Cerebrovascular Diseases.

[40] Dohle C, Pullen J, Nakaten A, Kust J, Rietz C (2009). Mirror therapy promotes recovery from severe hemiparesis: a randomized controlled trial. Neurorehabilitation and Neural Repair.

[41] Michielsen ME, Selles RW, van der Geest JN, Eckhardt M, Yavuzer G (2011). Motor recovery and cortical reorganization after mirror therapy in chronic stroke patients: a phase II randomized controlled trial. Neurorehabilitation and Neural Repair.

[42] Deconinck FJA, Smorenburg ARP, Benham A, Ledebt A, Feltham MG (2015). Reﬂections on mirror therapy: a systematic review of the effect of mirror visual feedback on the brain. Neurorehabilitation and Neural Repair.

[43] Külünkoğlu B, Erbahçeci F, Alkan A (2019). A comparison of the effects of mirror therapy and phantom exercises on phantom limb pain. Turkish Journal of Medical Sciences.

